# Chondroitin sulfate is required for follicle epithelial integrity and organ shape maintenance in *Drosophila*

**DOI:** 10.1242/dev.201717

**Published:** 2023-09-11

**Authors:** Collin Knudsen, Woo Seuk Koh, Tomomi Izumikawa, Eriko Nakato, Takuya Akiyama, Akiko Kinoshita-Toyoda, Greg Haugstad, Guichuan Yu, Hidenao Toyoda, Hiroshi Nakato

**Affiliations:** ^1^ Department of Genetics, Cell Biology, and Development, University of Minnesota at Twin Cities, Minneapolis, MN 55455, USA; ^2^ Faculty of Pharmaceutical Sciences, Ritsumeikan University, Shiga 525-8577, Japan; ^3^ Stowers Institute for Medical Research, Kansas City, MO 64110, USA; ^4^ Characterization Facility, University of Minnesota at Twin Cities, Minneapolis, MN 55455, USA

**Keywords:** Chondroitin sulfate, Ovary, Basement membrane, Muscle sheath, Chsy, *Drosophila*

## Abstract

Heparan sulfate (HS) and chondroitin sulfate (CS) are evolutionarily conserved glycosaminoglycans that are found in most animal species, including the genetically tractable model organism *Drosophila*. In contrast to extensive *in vivo* studies elucidating co-receptor functions of *Drosophila* HS proteoglycans (PGs), only a limited number of studies have been conducted for those of CSPGs. To investigate the global function of CS in development, we generated mutants for *Chondroitin sulfate synthase* (*Chsy*), which encodes the *Drosophila* homolog of mammalian chondroitin synthase 1, a crucial CS biosynthetic enzyme. Our characterizations of the *Chsy* mutants indicated that a fraction survive to adult stage, which allowed us to analyze the morphology of the adult organs. In the ovary, *Chsy* mutants exhibited altered stiffness of the basement membrane and muscle dysfunction, leading to a gradual degradation of the gross organ structure as mutant animals aged. Our observations show that normal CS function is required for the maintenance of the structural integrity of the ECM and gross organ architecture.

## Teaser

**Summary:** Chondroitin sulfate depletion results in muscle dysfunction and disrupted mechanical properties of the basement membrane, leading to age-dependent morphological defects in *Drosophila*.

## INTRODUCTION

Proteoglycans (PGs) are a special class of carbohydrate-modified proteins that have glycosaminoglycan (GAG) chains covalently attached to the core protein. Heparan sulfate (HS) and chondroitin sulfate (CS) are the most evolutionarily conserved GAGs, and are found in most animal species, from *C. elegans* and *Drosophila* to humans. It has been well established that HSPGs function as co-receptors for growth factor signaling, regulating distribution and reception of secreted signaling molecules (Esko and Selleck, 2002; Kirkpatrick and Selleck, 2007; Li and Kusche-Gullberg, 2016; Lindahl and Li, 2009; Xu and Esko, 2014). Genetic studies using the *Drosophila* model have helped establish roles of HSPGs in morphogen signaling (Nakato and Li, 2016) and in stem cell control (Bowden and Nakato, 2021). In *Drosophila*, HS-dependent secreted factors include FGFs, Decapentaplegic (Dpp; a *Drosophila* BMP), Wingless (Wg; a *Drosophila* Wnt), Hedgehog (Hh) and Unpaired (Upd; a ligand of the Jak/Stat pathway) (Nakato and Li, 2016).

In contrast to extensive *in vivo* studies elucidating the co-receptor functions of HSPGs, only a limited number of studies have been conducted for those of *Drosophila* CSPGs. This is partly because an *in vivo* model to systematically study the functions of CSPGs in development is lacking. We have previously identified a novel *Drosophila* CSPG: Windpipe (Wdp) (Takemura et al., 2020). Wdp is a single-pass transmembrane protein containing three GAG chains and leucine-rich repeat motifs, and is a previously unknown regulator of morphogen signaling pathways (Ren et al., 2015; Takemura et al., 2020). Given the structural similarity between HS and CS, one role of CSPGs appears to be the control of chemical signaling by modulating HS-dependent factors. On the other hand, CS is known to bind to many ECM components, including type I collagen, and plays a key structural role to orchestrate the ECM network (Ruoslahti, 1988). CS also interacts with water molecules, controlling osmotic pressure and hydration in the extracellular milieu (Comper and Laurent, 1978). Therefore, CS controls mechanical properties of the ECM and, thus, morphogenesis (Hwang et al., 2003; Lane et al., 1993; Mizuguchi et al., 2003). Such roles of CSPGs as ECM structural components and mechanotransduction regulators during *Drosophila* development remain to be determined.

Wdp is one of the few CSPG molecules that have been identified and investigated in *Drosophila*, whereas more than 25 are known in *C. elegans* (Noborn et al., 2018). This suggests that many *Drosophila* CSPGs remain to be discovered. However, CSPG core proteins are not well-conserved between species (Olson et al., 2006), and the identification of CSPGs cannot rely on the sequence homology to mammalian counterparts. It is therefore important to define the global function of CS using mutant animals that lack a component of the CS biosynthetic pathway.

In mammals, the polymerization of CS is achieved by enzyme complexes composed of multiple proteins that function redundantly (Mikami and Kitagawa, 2013). Chondroitin synthase 1 (Chsy1) is a key enzyme of these polymerase complexes. Owing to this redundancy, *Chsy1* knockout mice are viable, although CS production is reduced (Wilson et al., 2012). In humans, loss of *CHSY1* causes Temtamy preaxial brachydactyly syndrome, of which major features include limb malformations, short stature, hearing loss and delayed motor and mental development (Li et al., 2010; Tian et al., 2010). In *C. elegans*, where there is less redundancy in the CS polymerases, this gene is known as *squashed vulva 5* (*sqv5*), because its loss of function disrupts the invagination of the vulval epithelium (Herman et al., 1999). In addition to the vulval morphogenesis, the mutants show a defect in the progression of cytokinesis in early embryos, abnormal distal tip cell migration and reduced levels of chondroitin (Mizuguchi et al., 2003; Suzuki et al., 2006).

In this study, to determine the roles of CS during *Drosophila* development and homeostasis, we generated mutants for *Chsy*, the *Drosophila* homolog of mammalian Chsy1. Detailed analyses using the ovary as a model system revealed that CS is required for normal stiffness of the BM and the function of the ovarian muscle sheath. As a result, *Chsy* mutants show a gradual decay of the gross organ structure in an age-dependent manner, indicating that normal synthesis of CS is required for the maintenance of BM integrity and organ shape maintenance during aging.

## RESULTS

### Generation of *Chsy* mutant: a novel CS-deficient animal model

**Fig. 1.**
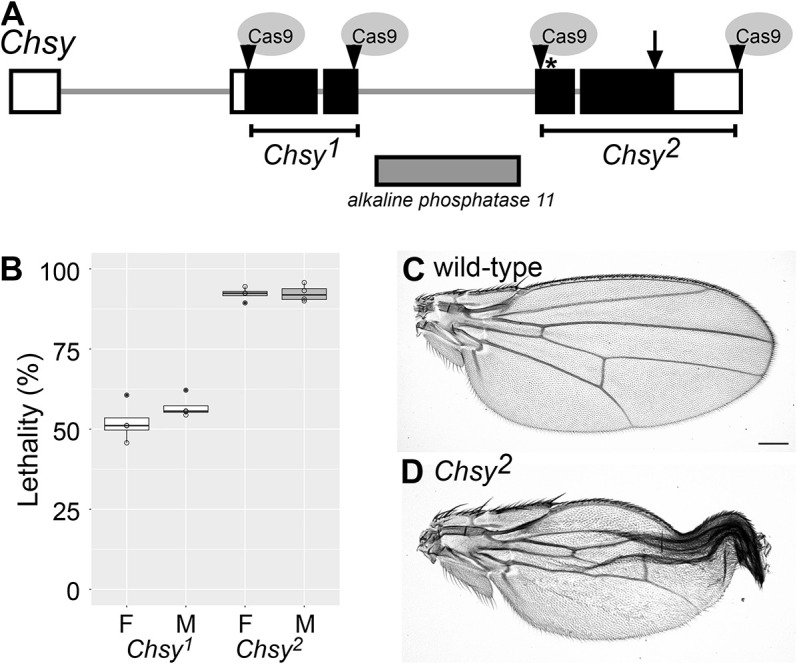
**Organization of the *Chsy* locus and generation of *Chsy* mutant alleles.** (A) A schematic of the *Chsy* locus and the generation of two mutant alleles, *Chsy^1^* and *Chsy^2^*, using the CRISPR-Cas9 system. Deletions of 973 bp (*Chsy^1^*) and 2050 bp (*Chsy^2^*) were made on either side of intronic *alkaline phosphatase 11* gene. The positions of Cas9 target sites are marked by arrowheads. The arrow indicates the position of the enzyme catalytic site; the asterisk indicates the position of a methionine residue. (B) Lethality of *Chsy^1^* and *Chsy^2^* alleles. The lethality rate of female homozygotes (F) or male hemizygotes (M) for *Chsy^1^* and *Chsy^2^* were calculated by three independent experiments. Four sets of experiments were performed for each sex; over 250 flies per set were counted. Boxes indicate the 25th and 75th percentiles; the median is marked with a line. The whiskers extend to the highest and lowest values within 1.5 times the interquartile range. (C,D) Adult wings are shown for wild-type (C) and *Chsy^2^* mutant (D) females. *Chsy* mutants show a wing maturation defect. Scale bar: 200 μm.

To investigate the global function of CS in development, we generated mutants for *Chondroitin sulfate synthase* (*Chsy*) using CRISPR-Cas9 mutagenesis. *Chsy* encodes the *Drosophila* homolog of human chondroitin synthase 1 (CHSY1). Owing to the presence of another gene (*alkaline phosphatase 11*) within the third intron of *Chsy*, we designed two different deletions (*Chsy^1^* and *Chsy^2^*), one on each side (Fig. 1A). Both mutations remove a large region of the coding sequence but do not affect expression of the intronic *alkaline phosphatase 11* gene (Fig. S1). The *Chsy^2^* allele removes the enzyme catalytic site (arrow in Fig. 1A) and is thus considered to be a null allele.

As a first step to characterize the *Chsy* mutant alleles, we conducted a lethality analysis. We found that the *Chsy^2^* allele revealed significantly higher lethality than *Chsy^1^* (Fig. 1B). This result suggests the possibility that the *Chsy^1^* allele may not be a null. We will discuss this later.

Adult survivors of both alleles show a folded wing phenotype. The distal region of a typical affected wing remains folded while the proximal part is partially expanded (Fig. 1D). Interestingly, wing patterning, which can be observed in unfolded wings, does not show any obvious abnormality. The penetrance of the wing phenotype of *Chsy^1^* and *Chsy^2^* was ∼72-79% in both sexes. These observations show that *Chsy* mutants show a defect in the wing maturation process, which is the last step of wing development (Bilousov et al., 2012; Kiger et al., 2001, 2007), but the wing patterning is not disturbed with *Chsy* mutations.

Specificity of these *Chsy* mutant phenotypes was examined using *Df(1)BSC707*, a deficiency line lacking the *Chsy* locus. We observed that *Df(1)BSC707*/*Chsy^2^* females show lethality at a comparable level with *Chsy^2^* homozygous females (91.2%, *n*=308). In addition, *Df(1)BSC707*/*Chsy^2^* adult wings exhibit a folded wing phenotype that is indistinguishable from that of *Chsy^2^* homozygotes (penetrance: 74.1%). The similar lethality rate and penetrance of the wing phenotype between *Df(1)BSC707*/*Chsy^2^* and *Chsy^2^*/*Chsy^2^* confirmed the amorphic nature of this allele.

### CS polymerization is disrupted in *Chsy* mutants

**Fig. 2.**
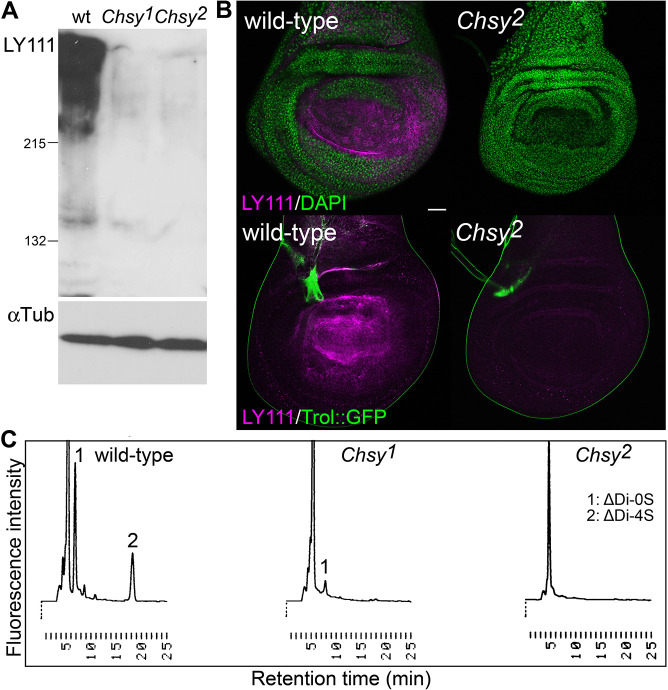
**CS polymerization is disrupted in *Chsy* mutants.** (A) Immunoblot analysis of *Drosophila* CSPGs. Protein extracts from wild-type, *Chsy^1^* and *Chsy^2^* adult flies were subjected to immunoblot analysis using anti-CS antibody (LY111). Anti-αTubulin antibody was used as the internal control. (B) Wing discs from wild-type (left) and *Chsy^2^* mutant (right) larvae were stained using anti-CS (LY111, magenta). In the top panels, discs were counterstained with DAPI (green). In the bottom panels, *trol-GFP*, a BM marker, outlines the wing discs (green). (C) CS disaccharide analysis. Chromatograms of unsaturated disaccharides from wild-type (left), *Chsy^1^* (middle) and *Chsy^2^* (right) mutant adult flies. After CS was completely digested with chondroitinase ABC, the resultant disaccharide species were separated by reverse-phase ion-pair chromatography (Docosil C22) with a post-column detection system. Peaks for the two disaccharides ΔDi-0S (1) and ΔDi-4S (2) are shown. Images are representative of 10-20 wing discs. All experiments in this figure used both female and male animals. Scale bar: 50 μm.

To determine whether CS production is affected in *Chsy* mutants, we performed immunoblot analysis using anti-CS antibody (LY111). This antibody detected high molecular weight CSPGs (>200 kDa) as smear bands in wild-type protein extracts (Fig. 2A) (Koh et al., 2023). These smear bands were undetectable in extracts prepared from *Chsy^1^* and *Chsy^2^* mutant alleles. The absence of the LY111 epitope in *Chsy* mutants was also confirmed by immunohistochemistry. In wild-type wing discs, the LY111 signal was detected broadly throughout the wing disc (Fig. 2B, left panels). CS is mainly localized on the basal side of the wing epithelium, largely overlapping with the basement membrane (BM) (Koh et al., 2023). In contrast, the LY111 staining was completely abrogated in the *Chsy* mutant discs (Fig. 2B, right panels).

We further examined detailed structure of CS isolated from the mutants by CS disaccharide analysis (Fig. 2C). Briefly, CS was purified from wild-type and *Chsy* mutant adult flies, and completely digested into disaccharides by chondroitinase ABC. The resultant disaccharide species, ΔDi-0S and ΔDi-4S, were separated and quantified by reversed-phase ion-pair chromatography with a post-column detection system (Toyoda et al., 2000). We found that the CS disaccharides were completely undetectable in *Chsy^2^*, indicating that this mutant allele essentially abolishes CS polymerization. On the other hand, we detected residual ΔDi-0S disaccharide but not ΔDi-4S in *Chsy^1^*.

Consistent with the lethality assay data, this observation supports the idea that *Chsy^2^* is a null allele, but *Chsy^1^* is not. We found that there is a methionine residue in frame in the fourth exon (asterisk in Fig. 1A). Therefore, a mRNA from the *Chsy^1^* locus can still encode a truncated protein with the enzyme catalytic site, which may have residual activity. These results suggest that *Chsy^1^* is a strong hypomorphic allele. Hereafter, we focused on *Chsy^2^* in the following analyses, which is simply referred to as *Chsy*. Together, these results show that *Chsy* is essentially required for CS chain elongation. The *Chsy* mutant alleles represent the first CS-deficient animal model in *Drosophila*.

### *Chsy* mutants show a defect in reproductive ability and display developmental delay

**Fig. 3.**
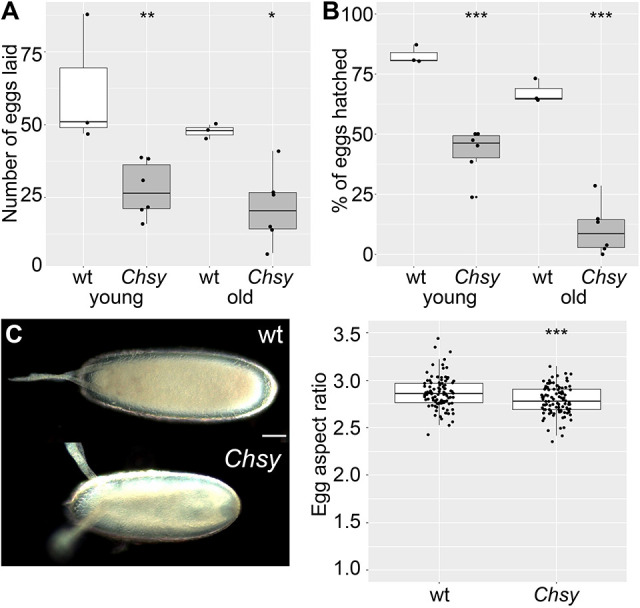
**A defect in reproductive ability and abnormal egg shape in *Chsy* mutants.** (A) Quantification of the average number of total eggs laid by 25 females over a 3 h period for young and old wild type and *Chsy* mutants (*Chsy*). Females at day 3 and day 21 after eclosion were used as young and old animals, respectively, in this and the following experiments. (B) Quantification of the proportion of eggs hatched for each respective genotype and age. Over 150 eggs were collected in total for the analysis of all genotypes and age, and were used for the subsequent analysis of egg quality. (C) In the left image, examples are shown for eggs laid by wild-type (top) and *Chsy* mutant (bottom) adults. The graph on the right shows the quantification of egg aspect ratio for each genotype. The egg aspect ratio was measured as egg length divided by egg width for each egg. Boxes indicate the 25th and 75th percentiles; the median is marked with a line. The whiskers extend to the highest and lowest values within 1.5 times the interquartile range. *Chsy* represents *Chsy^2^* in all panels. Scale bar: 50 μm. *P<0.05; **P<0.01; ***P<0.001 (two-sided, unpaired *t*-test).

As *Chsy* mutants show a high level of lethality, we next sought to determine their ability to reproduce. As shown in Fig. 3A, the number of eggs laid by the *Chsy* mutant females (day-3 after eclosion, young) is significantly smaller than wild-type control, indicating that *Chsy* mutants show a defect in reproductive ability. We found that the egg production was even more severely impaired in old females (day 21 after eclosion). Moreover, the proportion of eggs that hatched was also remarkably reduced in *Chsy* mutants, indicating that *Chsy* influences the quality of eggs laid (Fig. 3B). Importantly, the severity of this phenotype also prominently progresses with age: the hatching rate of eggs laid by old (day 21) females was drastically lower than that of young (day 3) females.

We noticed that eggs produced by *Chsy* mutant females were smaller and abnormal in shape. They are significantly shorter and less significantly thinner, resulting in a mild round egg phenotype with a slightly reduced length-to-width aspect ratio (Fig. 3C). The round egg phenotype is known to be caused by a defect in egg chamber elongation during oogenesis. It has been established that egg chamber elongation is achieved by a ‘molecular corset’ mechanism in which the ECM plays a crucial role (Bilder and Haigo, 2012; Cetera and Horne-Badovinac, 2015; Haigo and Bilder, 2011; Isabella and Horne-Badovinac, 2015). The high levels of female sterility, the reduced hatching and the abnormal egg shape suggest that oogenesis requires normal biosynthesis of CS.

### *Chsy* mutants show a defect of organ shape maintenance

**Fig. 4.**
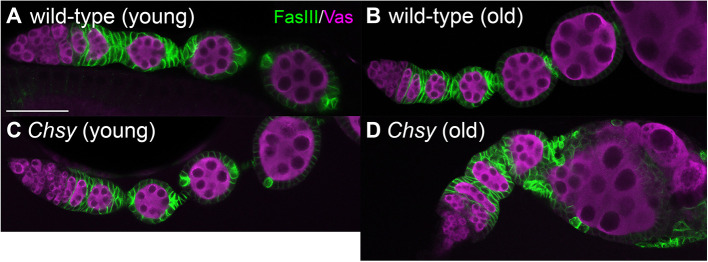
***Chsy* mutants show a defect in egg chamber shape maintenance.** (A-D) Ovarioles were dissected from wild-type (A,B) and *Chsy* mutant (C,D) *Drosophila* on day 3 (young; A,C) and day 21 (old; B,D) after eclosion. The samples were stained for FasIII (green, FCs) and Vas (magenta, germ cells). D shows an example of an old *Chsy* mutant ovariole with typical morphological defects. *Chsy* represents *Chsy^2^* in all panels. See Fig. 5A-F for more details. Images are representative of 20-31 ovarioles. Scale bar: 50 μm.

To study the function of *Chsy* during oogenesis, we analyzed the gross ovary morphology of *Chsy* mutants at different ages after eclosion (Fig. 4). The *Drosophila* ovary is composed of 16-20 ovarioles that contain progressively developing egg chambers. In wild type, oogenesis progresses with a proper organization of the growing egg chambers of 14 stages, each consisting of a germline cyst enclosed by a single layer of follicular epithelium, in a spatiotemporal order (Fig. 4A). Each egg chamber is separated by stalk cells – a type of differentiated follicle cell (FC). Ovaries from old (day 21 after eclosion) wild-type animals retained normal organ structures, indicating that no major age-dependent change occurs during this timeframe (Fig. 4B). The majority of young (day 3 after eclosion) *Chsy* mutant animals show no significant abnormality (Fig. 4C). In contrast, the ovariole morphology from aged (day 21) mutants was massively altered (Fig. 4D). In these ovarioles, individual egg chambers showed abnormal shape and lacked a spatiotemporally ordered alignment of oogenesis. Thus, *Chsy* mutants show a gradual decay of the gross organ structure in an age-dependent manner. Remarkably, we observed various distinctive morphological defects, as described below.

**Fig. 5.**
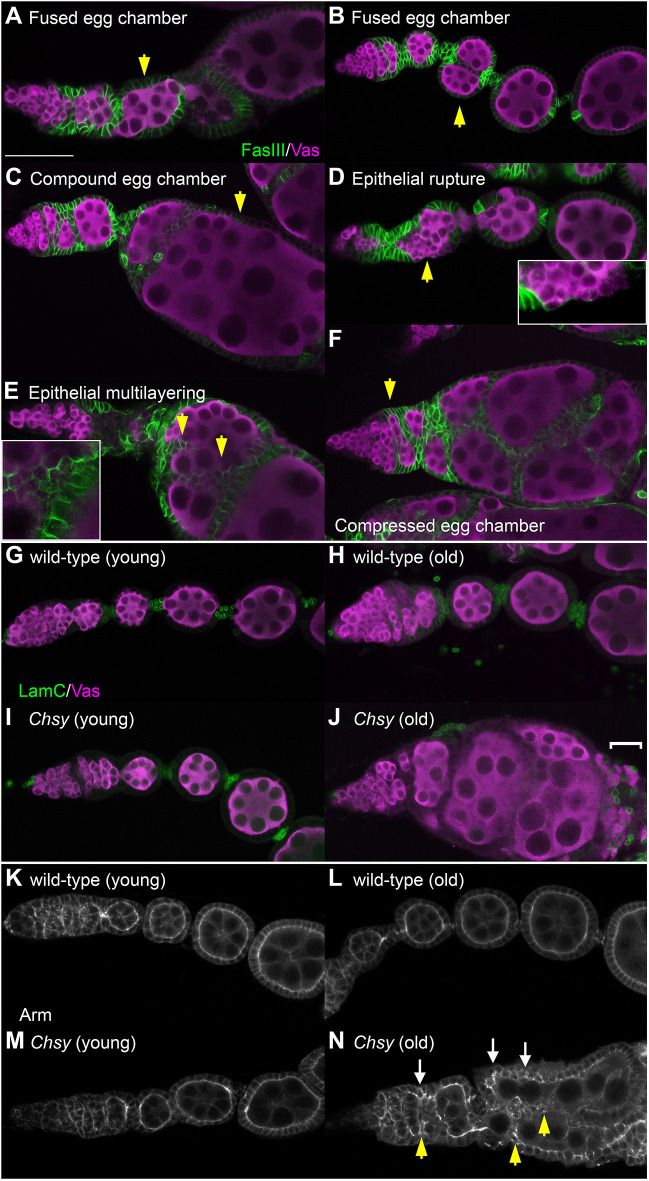
**Various morphological defects of aged *Chsy* mutant ovarioles.** (A-F) Six examples of old *Chsy* mutant ovarioles stained for FasIII (green, FCs) and Vas (magenta, germ cells). Each ovariole represents fused egg chamber (A,B), compound egg chamber (C), epithelial rupture (D), epithelial multilayering (E) and compressed egg chamber (F) phenotypes (arrowheads). For epithelial rupture (D) and epithelial multilayering (E), magnified views of the defects are provided in the insets. The penetrance of each phenotypic category observed in *Chsy* mutants at different ages is shown in Table 1. The ovariole shown in F was imaged together with that shown in Fig. 4D. (G-J) A stalk cell specification defect in *Chsy* mutant ovaries. Ovarioles were dissected from wild-type (G,H) and *Chsy* mutant (I,J) animals on day 3 (young; G,I) and day 21 (old; H,J) after eclosion. The samples were stained for LamC (green, stalk cells) and Vas (magenta, germ cells). Abnormal positioning of LamC-positive cells is marked by a bracket. The penetrance data of stalk cell specification defects in each genotype/age is shown in Table 2. (K-N) The apicobasal polarity of *Chsy* mutant follicular epithelia. Ovarioles were dissected from wild-type (K,L) and *Chsy* mutant (M,N) animals on day 3 (young; K,M) and day 21 (old; L,N) after eclosion. The samples were stained for Arm. Examples for abnormal AB polarity of FCs and the ingression of FCs into the cysts are marked by white arrows and yellow arrowheads, respectively. The penetrance data of abnormal Arm distribution in each genotype/age is shown in Table 2. *Chsy* represents *Chsy^2^* in all panels. Images are representative of 20-31 ovarioles. Scale bar: 25 μm.

**Table 1.**
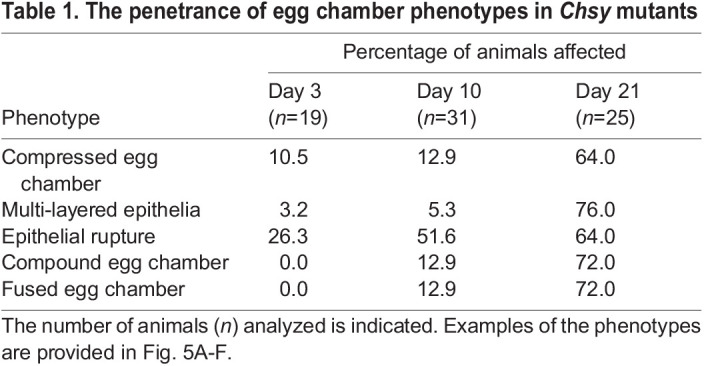
The penetrance of egg chamber phenotypes in *Chsy* mutants

**Table 2.**
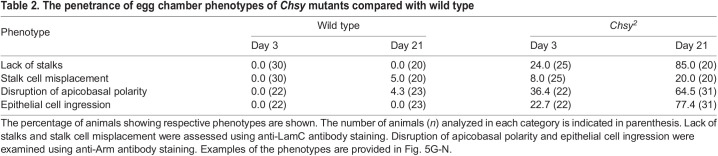
The penetrance of egg chamber phenotypes of *Chsy* mutants compared with wild type

We have previously shown that the loss of HS biosynthetic genes (e.g. *sulfateless*) in developing FCs disrupts Upd signaling, leading to the lack of stalks (Hayashi et al., 2012). We found that *Chsy* mutants also showed the loss of stalks (Fig. 5A). This results in the ‘fused egg chamber’ phenotype in which two or more egg chambers fail to be separated. As a direct result of the loss of stalk cells, egg chambers are expected to fuse along the anterior-posterior (AP) axis (Fig. 5A). Interestingly, however, *Chsy* mutant ovarioles often exhibit the fusion of two egg chambers aligned in an angle perpendicular to the AP axis (Fig. 5B). A previous study showed that 65% of the genes causing fused egg chambers also induced a ‘compound egg chamber’ phenotype (Berns et al., 2014). This phenotype is characterized by impaired cyst separation, in which one epithelial monolayer encloses two or more germline cysts. In fact, *Chsy* mutant ovarioles produce the compound egg chambers (Fig. 5C).

A possible cause of the transition from the fused egg chambers to the compound egg chambers is the disruption of the epithelial sheet that encloses each germline cyst. We indeed observed that *Chsy* mutants show the ‘epithelial rupture’ phenotype (Berns et al., 2014; Chanet and Huynh, 2020; Hongay and Orr-Weaver, 2011). In affected ovarioles, there were gaps of follicular epithelium where the cyst is not covered by epithelial cells (Fig. 5D). This observation showed that the loss of CS results in compromised epithelial integrity. Interestingly, we observed another type of epithelial abnormality in *Chsy* mutant egg chambers – ‘epithelial multilayering’ – in which a germline cyst is covered by two or more epithelial layers, instead of the normal single layer (Fig. 5E). In some cases, the epithelial cell clusters intruded into the germline cyst (yellow arrowheads in Fig. 5E).

In addition to the phenotypes with abnormal egg chamber organizations described above, the shape of individual egg chambers is severely altered in *Chsy* mutants. In wild-type, the egg chambers undergo stereotypic shape change. Early-stage egg chambers are spherical in shape, which then lengthen along the anterior-posterior (AP) axes into the oval shape as they mature into later stages. This egg chamber elongation requires proper synthesis, secretion and modifications of the BM components (Cetera and Horne-Badovinac, 2015; Horne-Badovinac et al., 2012; Isabella and Horne-Badovinac, 2016; Lewellyn et al., 2013). In aged *Chsy* mutants, the egg chambers change shapes in various manners, and they often elongate perpendicularly to the AP axis. This results in flattened ellipsoid egg chambers with a short AP axis radius, yielding a ‘compressed’ egg chamber phenotype (yellow arrowhead, Fig. 5F). In addition, most *Chsy* mutant ovarioles are characterized by a loss of at least one or more mid-stage egg chambers.

Importantly, the penetrance of all these phenotypes is low at the time of eclosion but increases as animals age (Table 1). Thus, in *Chsy* mutants, the ovariole formation occurs relatively normally, but the incidence of malformation dramatically increases during aging.

### *Chsy* is required for the proper specification of the stalk cell fate

We further analyzed the stalk cell specification in *Chsy* mutant ovarioles using an antibody against Lamin C (LamC), a marker for differentiated stalk cells (Borensztejn et al., 2018; Pearson et al., 2016). Anti-LamC antibody staining revealed normal stalk formation between individual egg chambers in wild-type (Fig. 5G,H) as well as young *Chsy* mutant ovarioles (Fig. 5I). In contrast, 85% of aged (day 21) *Chsy* mutant ovarioles show a defect in the proper specification of stalks, lacking one or more stalks (Fig. 5J). This is typically accompanied with fused or compound egg chambers (Fig. 5A-C,J; Berns et al., 2014; Hayashi et al., 2012).

Interestingly, *Chsy* mutants exhibited not only the loss but also a misplacement of stalk cells. In 20% of the aged mutant ovarioles, LamC-positive cells appeared at random locations in and around the egg chambers and failed to assemble the normal string-like configuration (bracket in Fig. 5J). As LamC expression is characteristic of cells that are subject to strong mechanical stress (Donohoe et al., 2018), this may be associated with altered mechanical properties of the BM in *Chsy* mutant ovarioles, as we discuss below.

### Apicobasal polarity and adherens junction organization are disrupted in *Chsy* mutants

The epithelial multilayering phenotype has previously been observed in mutants that affect adherens junctions (AJs) and/or the apicobasal (AB) cell polarity (Berns et al., 2014; Bilder et al., 2000; Harris and Tepass, 2008). To analyze the AJ organization and the AB polarity in *Chsy* mutants, we used an antibody against Armadillo (Arm). *arm* encodes the *Drosophila* β-catenin, a key component of the AJs. In wild type and most young *Chsy* mutants, Arm was properly localized near the boundary of FCs and germline cysts (Fig. 5K-M). In contrast, we detected Arm on the basolateral side of FCs in aged *Chsy* mutants, indicating that the AB polarity of FCs was disrupted (white arrows, Fig. 5N). In some cases, Arm was localized between the nurse cells inside the germline cysts, instead of the FC-germline boundaries (yellow arrowheads, Fig. 5N). A previous report showed that this phenotype was caused by ingression of epithelial cells into the cysts (Berns et al., 2014; Bilder et al., 2000; Chanet and Huynh, 2020), which we sometimes observed in *Chsy* mutant egg chambers (arrowheads in Fig. 5E). Similar to other phenotypes, the penetrance of this abnormality increased during aging (Table 2). Thus, *Chsy* is required for the maintenance of the AB polarity of the follicular epithelium.

### BM becomes morphologically disorganized in *Chsy* mutants

**Fig. 6.**
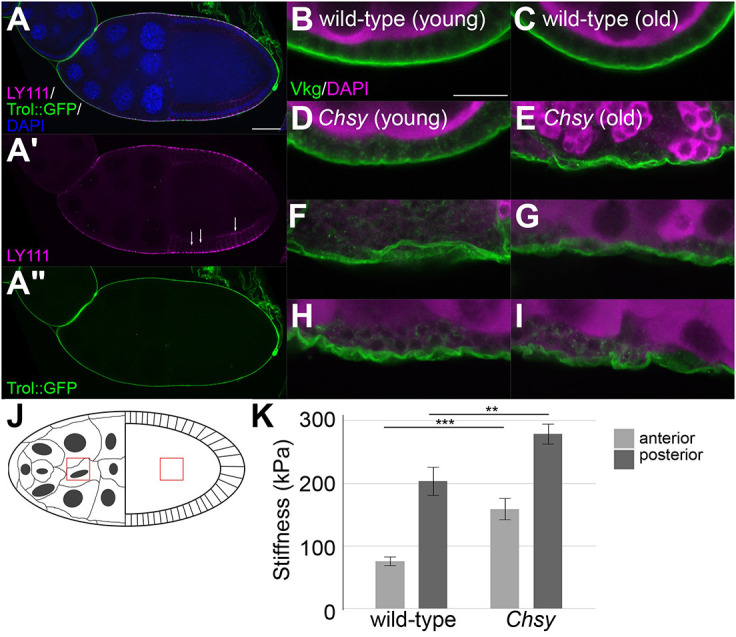
**Disorganized BM morphology and mechanical properties in *Chsy* mutants.** (A-A″) A stage 10 egg chamber bearing *trol::GFP* (green, A″) stained with anti-CS (LY111) antibody (red, A′). Blue shows DAPI staining (A). White arrows indicate puncta of LY111 signal (A′). (B-I) Ovarioles were dissected from wild-type (B,C) and *Chsy* mutant (D-I) animals on day 3 (B,D) or day 21 (C,E-I) after eclosion. Several examples are shown for aged *Chsy* mutants (E-I). The images in E and F represent different regions of the same ovariole; those in H and I represent different regions of a different ovariole. The samples were stained for Vkg (green, BM) and Vas (magenta, germ cells). (J) Schematic representation of stage 10 egg chamber with red boxes showing where the Young's modulus values were obtained in the anterior and posterior regions. Anterior (nurse cells) is towards the left and posterior (oocyte) is towards the right. (K) BM Young's modulus (stiffness) from 3-day-old wild-type and *Chsy* mutants in the indicated anterior and posterior regions of stage 10 egg chambers (*n*=3 for wild type and *Chsy*). Data are mean±s.e.m. *Chsy* represents *Chsy^2^* in all panels. Images are representative of 10-20 ovarioles. Scale bars: 50 μm in A; 10 μm in B. **P<0.01; ***P<0.001 (two-sided, unpaired *t*-test).

We analyzed the distribution of CS in the ovary using anti-CS antibody (LY111). The LY111 signal was mainly detected in the BM layer visualized by a protein trap line of Perlecan, *trol-GFP* (Fig. 6A). The LY111 epitope outlined the entire egg chamber, largely overlapping with the BM, and some intense signals were detected as puncta (white arrows in Fig. 6A′). The nature of these intense signals, however, is unknown.

To determine whether CS depletion affects the BM morphology, *Chsy* mutant ovarioles were stained with antibody against Viking (Vkg), the α2 chain of the *Drosophila* ColIV (Van De Bor et al., 2021). In the wild-type ovarioles, normal formation of thin BM was observed at all ages (Fig. 6B,C). Similarly, young *Chsy* mutant ovarioles did not show any aberrant BM structures (Fig. 6D). In contrast, as *Chsy* mutant animals aged, the BM became thickened and morphologically disorganized, with numerous gaps within the layers, accompanied by alterations in the gross architecture of ovarioles (Fig. 6E-I). Together, our results suggest that the initial assembly of the organ can occur in the absence of *Chsy* but that normal CS biosynthesis is required for structural integrity of the BM and organ architecture in aged animals.

### CS is required for normal mechanical properties of the BM

Many cells are known to sense rigidity of the ECM via mechanotransduction and change their behaviors (Charras and Sahai, 2014). Therefore, one possible mechanism for the failure of organ shape maintenance in *Chsy* mutants is an altered physical environment of the ECM. To examine the effect of CS depletion on mechanical properties of tissues, we measured BM stiffness (Young's modulus) in live *Chsy* mutant ovarioles using atomic force microscopy (AFM) (Chen et al., 2017; Chlasta et al., 2017; Crest et al., 2017; Lamb et al., 2021). Following the recently established protocol (Topfer et al., 2022a), 3-day-old wild-type and *Chsy* mutant ovaries were freshly dissected in a tissue culture medium and their stiffness was measured at anterior (nurse cell regions) and posterior (oocyte regions) locations in stage 10 egg chambers (Fig. 6J). Wild-type posterior regions were significantly stiffer than anterior regions, consistent with previous studies (Chlasta et al., 2017; Crest et al., 2017) (Fig. 6K). Importantly, the average stiffnesses of both regions in *Chsy* mutants were significantly larger than the equivalent areas in wild type (Fig. 6K). It is interesting that CS depletion makes the BM stiffer, as losing BM components more commonly results in a decrease in stiffness (Chlasta et al., 2017). However, there are some examples of an increased stiffness in BM component mutants (Topfer et al., 2022b). Taken together, CS is required for normal mechanical properties of the BM. The altered BM stiffness in *Chsy* mutants likely contributes to the organ maintenance defect.

### Morphology and function of muscle sheath in *Chsy* mutant ovaries

Several lines of evidence suggested a possibility that *Chsy* affects muscle development and function. First, a *Drosophila* CSPG, Kon-tiki (Kon), is required for muscle-tendon attachment in embryonic muscles (Estrada et al., 2007; Perez-Moreno et al., 2017; Schnorrer et al., 2007), as well as in the adult flight and abdominal muscles (Perez-Moreno et al., 2014; Weitkunat et al., 2014). In mammals, CS plays a crucial role in muscle formation and pathogenesis (Mikami et al., 2012; Negroni et al., 2014; Snow et al., 2005; Takeuchi et al., 2016). Furthermore, *Chsy* mutant phenotypes, including the reduced egg aspect ratio and compressed egg chambers, closely resemble defects previously observed in mutant animals with a disrupted ovarian muscle sheath that surrounds developing egg chambers (Andersen and Horne-Badovinac, 2016).

**Fig. 7.**
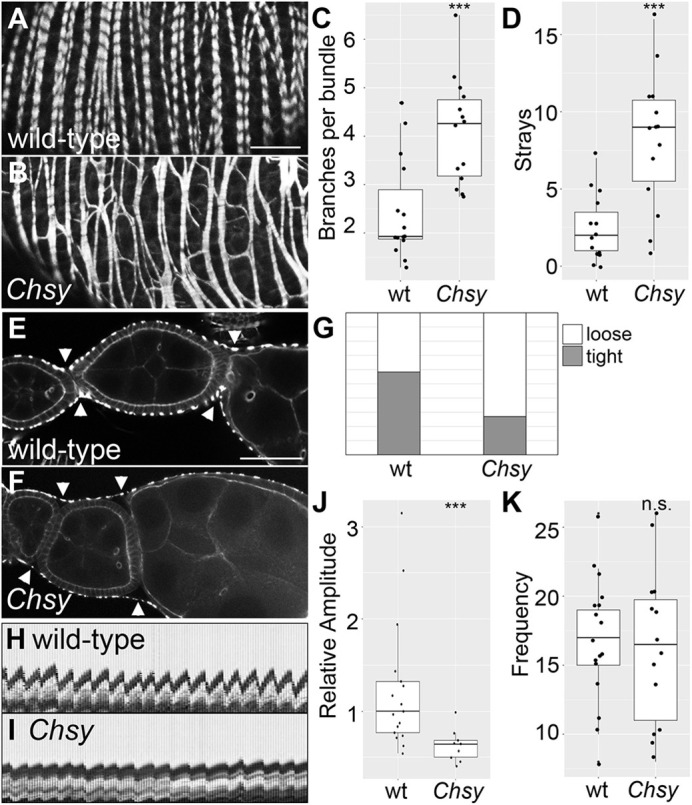
**Muscle sheath morphology and function in *Chsy* mutant ovaries.** (A-D) Stage 7-8 egg chambers of day 4 wild-type (A) and *Chsy* mutant (B) ovaries were stained with phalloidin to visualize the ovarian muscle sheath. (C,D) The average number of branches per sarcomere bundle (C) and the number of ‘stray’ sarcomeres (D). A ‘stray’ sarcomere is defined as one that deviates from the major sarcomeres, which lay perpendicular to the AP axis of the egg chamber, by an angle of 45° or more. (E-G) Muscle sheath tightness assay. Muscle sheath from wild-type (E) and *Chsy* mutant (F) ovaries was visualized using phalloidin staining. Muscle sheath morphology was classified into two groups: tight and relaxed. In tight samples, the muscle sheath closely outlines egg chambers. In contrast, there are gaps between egg chambers and the muscle sheath evident in the bridge area in the relaxed examples (compare arrowheads in E and F). The ratio of tight and relaxed samples in each genotype (*n*=24 for wild-type; *n*=26 for *Chsy*) is shown in G. (H-K) A muscle sheath contraction defect in *Chsy* mutant ovaries (*n*=17 for wild-type; *n*=14 for *Chsy*). Live imaging was used to determine whether *Chsy* affects muscle sheath function. Representative kymographs showing the contraction patterns of the muscle sheath from wild-type (H) and *Chsy* mutant (I) ovaries. For live images, see Movie 1 (wild type) and Movie 2 (*Chsy* mutant). Quantification of the amplitude (J) and the frequency (K) of the contraction is shown. Boxes indicate the 25th and 75th percentiles; the median is marked with a line. The whiskers extend to the highest and lowest values within 1.5 times the interquartile range. *Chsy* represents *Chsy^2^* in all panels. Images are representative of 14-26 ovarioles. Scale bars: 20 μm. ***P<0.001; n.s., not significant (two-sided, unpaired *t*-test).

Each ovariole is surrounded by the epithelial muscle sheath, or the muscle sheath, composed of mononuclear muscle cells (Hudson et al., 2008). To determine whether *Chsy* affects muscle sheath morphology, we stained wild-type and *Chsy* mutant ovarioles with phalloidin. The major fibers of the muscle sheath run perpendicular to the AP axis of the ovariole (Fig. 7A). This pattern of circular fibers is disrupted in *Chsy* mutants, with significantly increased branching per sarcomere bundle (Fig. 7B,C). In addition, the number of branches that deviate from the major sarcomeres by an angle of 45° or greater, which we term ‘stray’ sarcomeres, is increased in the mutant (Fig. 7D). The branching and stray defects were confirmed with myosin staining (Fig. S2A,B). We also analyzed the ‘periodicity’, a spacing pattern of sarcomeres, by measuring space between anti-myosin antibody signals. The space between myosin signals, or sarcomere length, showed no difference between wild-type and *Chsy* mutants (Fig. S2C). However, the proportion of ovarioles that showed a loss of sarcomere periodicity is dramatically increased in *Chsy* mutants (Fig. S2D). We defined the loss of periodicity as when the anti-myosin staining showed a continuous signal (arrowheads in Fig. S2B). Furthermore, we also noticed that the muscle sheath in *Chsy* ovarioles tended to be ‘loose’: there is space between the egg chambers and the muscle sheath layer (Fig. 7E-G; Andersen and Horne-Badovinac, 2016).

The muscle sheath contraction contributes to the movement of the egg chambers posteriorly toward the oviduct. To examine the effect of CS depletion on the muscle function, we observed the contraction of *Chsy* mutant ovary in culture. Live imaging showed that cultured wild-type ovaries continued robust rhythmic contraction (Movie 1). In contrast, the ovary contraction was significantly weakened in *Chsy* mutant ovaries (Movie 2). As shown in representative kymographs (Fig. 7H,I) and quantifications (Fig. 7J,K), the loss of CS impaired the amplitude but not the frequency of the ovary contraction. This phenotype appears to be consistent with a disrupted BM-muscle linkage. Our analysis using anti-integrin β PS antibody did not detect any obvious defect in muscle-muscle linkage in the *Chsy* mutant ovary (Fig. S3). *Chsy* may disrupt the ovarian sheath muscle in an integrin-independent manner, or integrin may remain in the correct position but is dysfunctional. Further studies are required to determine the molecular basis for the muscle function abnormalities in *Chsy* mutants.

To determine whether *Chsy* mutation affects the ovarian muscle sheath specifically or also damages other muscle types, we examined the morphology of the adult indirect flight muscles (IFMs) using phalloidin staining. We found that *Chsy* mutants show myofibril abnormality in some areas of the hemithorax, with high amounts of muscle overlapping (Fig. S4A,B). This results in a loss of the horizontal sarcomere patterning found in wild-type dorsal longitudinal muscles, showing a ‘spaghetti’ phenotype. Our analysis using anti-Talin antibody staining did not detect any obvious abnormality in the myotendinous junction (Fig. S4C,D). Therefore, the molecular basis for the IFM myofibril phenotype of *Chsy* mutant adults is unknown at this point. These results, in conjunction with the ovary muscle defects, suggest that CS may be commonly required for the morphology, function and arrangements of muscles or myofibrils.

Taken together, these results show that CS is required for muscle sheath integrity and contractile activity. The altered BM mechanical properties and possible impairment of muscle anchorage, together with the impaired but continued contraction, may contribute to the progressive damage of the egg chamber architecture.

## DISCUSSION

The *Drosophila* ovary has been a powerful model system for studying the role of the BM during development and organ maintenance (Diaz-Torres et al., 2021; Haigo and Bilder, 2011; Isabella and Horne-Badovinac, 2015; Ramos-Lewis and Page-McCaw, 2019). The BM regulates organ development via different mechanisms. First, it affects signaling to contacting cells by binding or sequestering specific signaling ligands (Ma et al., 2017; Wang et al., 2008). Second, the BM provides a physical scaffold for tissue morphogenesis (Charras and Sahai, 2014; Chlasta et al., 2017; Isabella and Horne-Badovinac, 2015; Urbano et al., 2009; Chen et al., 2019; Crest et al., 2017; Topfer et al., 2022b).

Previous studies have revealed a variety of developmental abnormalities caused by disruptions of the BM components. For example, the failure of proper deposition of BM components disrupts egg chamber rotation and elongation, leading to production of round eggs (Haigo and Bilder, 2011; Isabella and Horne-Badovinac, 2016). An impairment of coordinated deposition of ColIV caused various defects, including loss and displacement of stalk cells, fused egg chambers, compound egg chambers and epithelial rupture (Van De Bor et al., 2021). We found that these morphological defects largely overlap with *Chsy* mutant phenotypes. The resemblance of the mutant phenotypes between *Chsy* and BM components, together with the observation that CS is mainly localized in the BM, supports the idea that the basis of various morphological abnormalities in *Chsy* lies mainly in the disrupted BM.

We observed that the initial assembly of the organ can occur relatively normally in the absence of CS, but normal *Chsy* function is required for the maintenance of the BM structural integrity and gross organ architecture. The mechanisms for the age-dependent phenotypes of *Chsy* mutants are not completely understood but may be explained at least in part by the two major consequences of CS depletion: disarrangement of BM stiffness and altered muscle sheath functioning and morphology. First, *Chsy* mutants show an increased stiffness in the BM of the egg chamber. Based on previous studies, this change in the mechanical properties is likely to affect epithelial integrity and behaviors, leading to the instability of organ shape (Crest et al., 2017; Haigo and Bilder, 2011; Isabella and Horne-Badovinac, 2016; Van De Bor et al., 2021). Second, the mutants also showed the disrupted morphology and function of muscle sheath surrounding the egg chambers. The reduced muscle contractility in *Chsy* mutants suggests an incompletely developed muscle-tendon attachment. This is consistent with the fact that a *Drosophila* transmembrane CSPG Kon mediates muscle-tendon adhesion (Perez-Moreno et al., 2014, 2017; Weitkunat et al., 2014). Although it is unknown whether Kon plays a role in the ovary, our observation suggests that a similar CSPG-dependent mechanism may regulate the linkage of the muscle sheath. Our anti-Talin staining did not reveal any gross defect in the myotendinous junction of IFMs in *Chsy* mutants (Fig. S4) and more-detailed analysis will be required to understand the role of CS in the muscle attachment. A previous study demonstrated that muscle sheath hypo-contraction resulted in progressive disruption of egg chamber organization and the production of rounded eggs (Andersen and Horne-Badovinac, 2016), which are similar to the *Chsy* phenotypes. With the continued muscle contraction, we speculate that the muscle bundles gradually detach from the egg chambers (contraction-induced damage), leading to the age-dependent loss of the epithelial integrity and organ shape degeneration. Thus, the functions of CS as an ECM scaffold and muscle receptor provide a reasonable model for the age-dependent phenotypes of *Chsy* mutants. Importantly, the stiffness and muscle functioning defects are observed in young animals before the ovariole morphology becomes abnormal. Thus, we propose that these early defects are at least partly responsible for the age-dependent phenotypes observed.

The loss of CS in *C. elegans* by mutations in *sqv-5*, the worm homolog of human ChSy-1 and *Drosophila Chsy*, causes defects in embryonic cell division and vulval morphogenesis (Hwang et al., 2003; Mizuguchi et al., 2003). *sqv-5* mutant hermaphrodites showed 50% lethality (Mizuguchi et al., 2003). The remaining 50% survived, probably due to the maternally supplied mRNAs as the progenies produced from the mutant hermaphrodites were completely lethal. It is important to note that all *Chsy* mutants we used in this study, except our lethality assay, were from the established homozygous mutant lines. Therefore, *Chsy^2^* mutants were both maternally and zygotically null. Given the complete lethality of sqv-5 ‘null’ mutants, it is striking that a fraction (∼5%) of *Chsy^2^* mutants survived to adult stages. CS biosynthesis requires two distinct *N*-acetylgalactosaminyltransferase (GalNAcT) activities: GalNAcT-I activity for the addition of the first GalNAc residue to the linkage tetrasaccharide and GalNAcT-II activity for chain elongation. Sqv-5 is unique as it catalyzes both reactions (Hwang et al., 2003; Mizuguchi et al., 2003). Our analyses showed that *Drosophila* Chsy is clearly required for the chain backbone polymerization but its function in the chain initiation is unknown. To further understand the mechanistic aspects of the *Chsy* mutant phenotypes, future studies are required to elucidate the exact enzymatic activity of Chsy in CS biosynthesis.

Previous studies on Wdp highlighted the similarity of the functions of this CSPG to the roles of HS in morphogen signaling (Ren et al., 2015; Takemura et al., 2020). On the other hand, *Chsy* mutants exhibited phenotypes that have never been observed in HS-related mutants, representing unique roles of CS. For example, *Chsy* mutations do not appear to have a significant impact on the wing patterning but normal biosynthesis of CS is required for the wing maturation process. This shows a striking contrast to the functions of HS in the wing patterning. Furthermore, the age-dependent breakdown of gross organ structure is also unique to *Chsy* mutants. Thus, phenotypic analyses of *Chsy* mutants, the first CS-deficient animal model in *Drosophila*, defined a few examples of the specificity of HS versus CS functions, and suggest that CS functions at the interface of chemical signaling and mechanotransduction during tissue morphogenesis and maintenance. Further studies are needed to define which biological phenomena/molecular networks are co-regulated by HS and CS, and which are controlled by either HS or CS.

## MATERIALS AND METHODS

### *Drosophila* strains

Oregon-R was used as a wild-type control. A deficiency line lacking the *Chsy* locus, *Df(1)BSC707*, was obtained from the Bloomington Drosophila Stock Center (BDSC, 26559). Flies were raised on a standard cornmeal fly medium at 25°C unless otherwise indicated.

*Chsy^1^* and *Chsy^2^* mutant strains were generated by CRISPR/Cas9-mediated nonhomologous end joining as previously described (Takemura et al., 2020). sgRNA sequences targeting *Chsy*, chosen using CRISPR Optimal Target Finder, were cloned into pU6-BbsI-chiRNA (a gift from Melissa Harrison, Kate O'Connor-Giles and Jill Wildonger, University of Wisconsin-Madison, USA). Combinations of two sgRNA-containing plasmids were injected into the yw; nos-Cas9(y+)/CyO strain by Genetivision to delete regions of the *Chsy* gene and repair by NHEJ. Resultant deletions (973 bp and 2050 bp for *Chsy^1^* and *Chsy^2^*, respectively) were screened via PCR, verified by Sanger sequencing, followed by backcrossing with Oregon-R strain for five generations. The following sgRNA sequences were used: sgRNA1 for *Chsy^1^*, 5'-GCTGAAGAACTACCTGGCGCTGG-3′; sgRNA2 for *Chsy^1^*, 5'-GAAAGCGGAGGAGATGCGTCAGG-3′; sgRNA3 for *Chsy^2^*, 5'-GCACCGACGACCTGCTGGAC-3′; sgRNA4 for *Chsy^2^*, 5'-GTTATCAACTCTCGATAGCT-3′.

### Preparation of adult wings

The right wings from female flies were dehydrated in ethanol and subsequently with xylene (Fujise et al., 2001). The specimens were mounted in Canada balsam (Benz Microscope).

### Immunohistochemistry and immunoblot analysis

Immunostaining of the ovaries and adult indirect flight muscles was performed as previously described (Hayashi et al., 2009, 2012; Lemke et al., 2019). Images were obtained using a Zeiss 710 laser scanning confocal microscope. The primary antibodies used were as follows: mouse anti-Fasciclin III 7G10 [1:50, Developmental Studies Hybridoma Bank (DSHB)], mouse anti-Lamin C LC28.26 (1:100, DSHB), rabbit anti-Vasa (1:50, a gift from S. Kobayash, University of Tsukuba, Japan), mouse anti-Armadillo-s N27A1(1:100, DSHB), rabbit anti-Viking (1:2000, a gift from S. Noselli, Université Côte d'Azur, France), mouse anti-Myosin heavy chain 3E8-3D3 (1:50, DSHB), mouse anti-integrin β PS CF.6G11 (1:10, DSHB), mouse anti-Talin A22A (1:10, DSHB) and mouse anti-CS A (1:100, Tokyo Chemical Industry, LY111). Alexa488-, Alexa568- and Alexa633-conjugated secondary antibodies (anti-mouse IgG 488, A11001; anti-mouse IgG 568, A-11031; anti-mouse IgG 633, A21050; anti-mouse IgM 568, A-21043; anti-rabbit IgG 488, A11008; anti-rabbit IgG 633, A21070; Thermo Fisher Scientific) were used at a dilution of 1:200. Alexa Fluor 633-Phalloidin (1:50, Thermo Fisher Scientific, A22284) was used to visualize the sheath muscle bundles. Egg chamber phenotypes (Tables 1 and 2) were quantified by counting the number of ovarioles exhibiting the particular phenotype and dividing by the total number of ovarioles examined.

For immunoblot analysis, protein samples were extracted from *Drosophila* adult flies by SDS sample buffer. Mouse anti-CS A (1:1000, Tokyo Chemical Industry, LY111) and mouse anti-αTubulin antibody (1:2000, Sigma-Aldrich, DM1A) were used as primary antibodies. Signals were detected using HRP-conjugated secondary antibodies and Pierce ECL Western Blotting Substrate (Thermo Fisher Scientific).

### Live imaging of ovary contractions

Whole ovaries were dissected in S2 tissue culture medium, and movies were captured using a Zeiss 710 microscope. Kymographs were generated using ImageJ.

### RT-PCR

Expression of the intronic gene *Alp11* was analyzed by RT-PCR. *Actin5C* was used as a control. As *Alp11* expression is high in the testis, total RNA was isolated from 20 adult testes of wild-type, *Chsy^1^* and *Chsy^2^* males. The tissues were homogenized in 300 μl of TRIzol Reagent (Invitrogen, 15596-026), and total RNA was isolated using Direct-zol RNA MiniPrep (Zymo Research, R2050). cDNA was synthesized from 50 ng of total RNA using SuperScript III First-Strand Synthesis System for RT-PCR (Invitrogen, 18080-051).

A 0.5 μl aliquot of the cDNA synthesis reaction mixture was used to amplify the target cDNAs using the following PCR primers: *Alp11* (forward), 5′-CAGCACCCTCAACTATGCCA-3′; *Alp11* (reverse), 5′-CTGGGTGTGTAGGATGTGGG-3′; *Act5C* (forward), 5′-GGCGCAGAGCAAGCGTGGTA-3′; *Act5C* (reverse), 5′-GGGTGCCACACGCAGCTCAT-3′*.* PCR products of expected size (102 bp and 124 bp, respectively) were analyzed by agarose gel electrophoresis.

### Disaccharide analysis

CS isolation and disaccharide composition analysis were carried out as previously described (Dejima et al., 2013; Kamimura et al., 2006; Kleinschmit et al., 2010; Nakato et al., 2019; Toyoda et al., 2000). Approximately 200 mg of adult flies were used to isolate CS. The CS sample was digested with chondroitinase ABC (1 mU; EC4.2.2.4, Seikagaku), and the resulting disaccharide species were separated using reverse-phase ion-pair chromatography [Docosil C22 (4.6×150 mm; particle size, 5 μm), Senshu Scientific]. The effluent was monitored fluorometrically for post-column detection of HS disaccharides (Toyoda et al., 2000).

### Atomic force microscopy (AFM)

BM stiffness (apparent Young's modulus) of stage 10 egg chambers was determined using AFM (Haugstad, 2012; Topfer et al., 2022a). Briefly, ovaries from 3-day-old *Drosophila* were freshly dissected in a tissue culture medium containing 15% fetal bovine serum with 600 U ml^−1^ penicillin and 600 U ml^−1^ streptomycin in M3 medium. Immediately before dissecting, insulin was added to a concentration of 0.2 mg ml^−1^. Isolated stage 10 egg chambers were adhered to poly-D-lysine-coated Petri dishes and recovered with the tissue culture medium. For each egg chamber, AFM measurements were taken on anterior and posterior regions equidistant from the poles. Three egg chambers were examined for both genotypes.

AFM-based nanomechanical analysis in the form of force-distance mapping was performed using a Keysight 5500 scanning probe microscope running Picoview 1.20 software. Data were collected with two silicon tips (nominal end radius of curvature ∼10 nm, cone half-angle of 20-25° over the final 1 μm of the tip) attached to backside Al-coated rectangular silicon cantilevers (NuNano type SCOUT 70), each with determined spring constant k=2.2±0.2 N/m (needed to calibrate force measurements). At least five ‘force volume datasets’ were acquired over distinct 10×10 μm subregions (8×8 grid of sites in each case) on the anterior and posterior regions of interest, using a closed loop X-Y scanner (model 9524). The approach-retract cycling period per force-distance curve was 500-600 ms and the ultimate loading force in each cycle was ∼25-30 nN. Z sensor data were used for linearization of the distance scale. Only the first 50 nm of indentation were used to determine BM modulus. Post-processing of the arrays of force-distance data was performed using a custom Matlab program, SPMLAB: a robust spectral data analysis software for scanning probe microscopy (Technology Commercialization, Technology No. 2021-291, University of Minnesota).

## Supplementary Material

10.1242/develop.201717_sup1 Supplementary information
