# Supplementary material for: Chondroitin sulfate is required for follicle epithelial integrity and organ shape maintenance in Drosophila
Source: Development. 2023 Sep 11;150(17):dev201717. doi: 10.1242/dev.201717 (PMC10508698; doi:10.1242/dev.201717)
Supplement: Supplementary information [file develop-150-201717-s1.pdf]

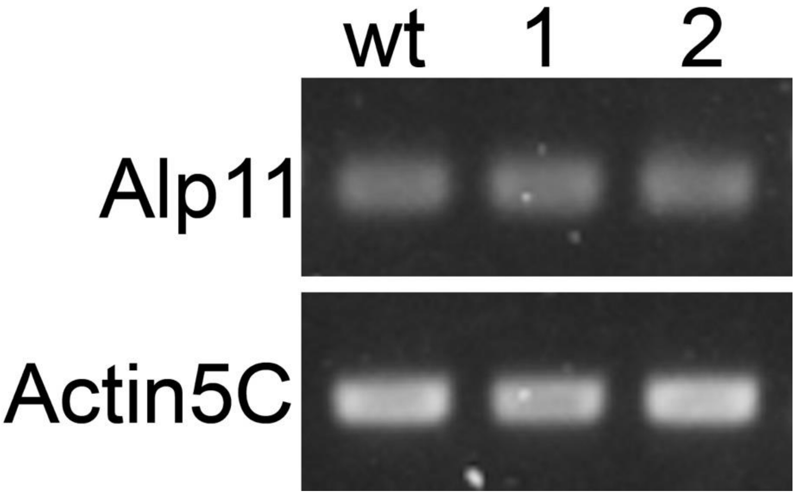

Fig. S1. *Alp11* expression in *Chsy* mutant alleles.
RT-PCR was performed to detect *alkaline phosphatase 11* (*Alp11*) transcripts in the testes from wild-type (wt), $Chsy^1$ (1), and $Chsy^2$ (2) mutants. Actin5C was used as an internal control. No obvious change in the *Alp11* expression level was detected in *Chsy* mutants.

Development • Supplementary information

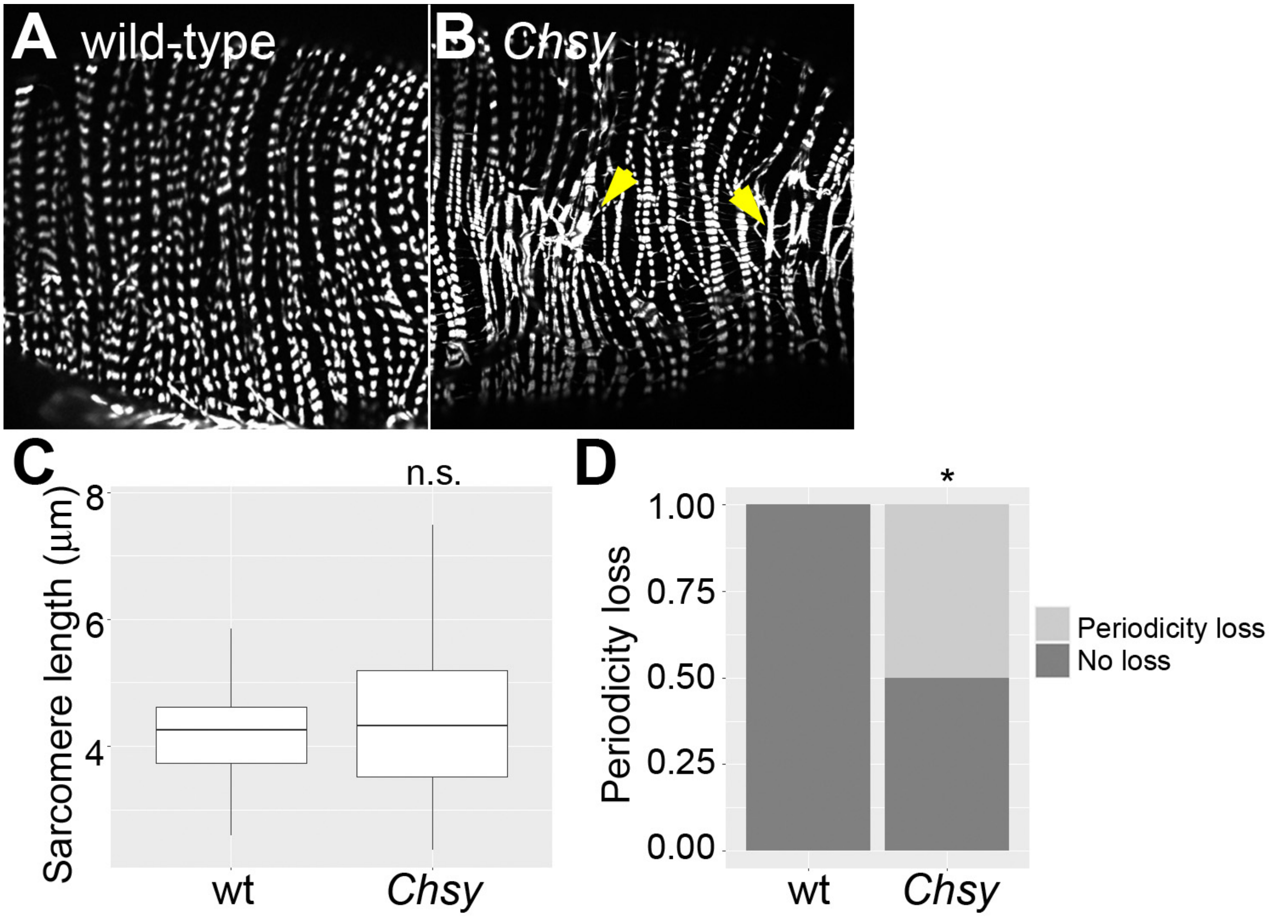

Fig. S2. Myosin staining of *Chsy* mutant egg chamber.

(A and B) Stage 7-8 egg chambers of day-4 wild-type (A) and *Chsy*^{2} mutant (B) ovarioles were stained with anti-Myosin antibody. (C and D) Sarcomere periodicity, a spacing pattern of sarcomeres, was analyzed by measuring the space between anti-Myosin antibody signals. There was no difference in sarcomere length quantified with anti-myosin staining between wild-type and *Chsy* mutants (C). The proportion of ovarioles showing a loss of periodicity is dramatically increased in *Chsy* mutant (D). The yellow arrowheads (B) show examples of sarcomere periodicity loss. n.s., not significant; *P<0.05.

Development • Supplementary information

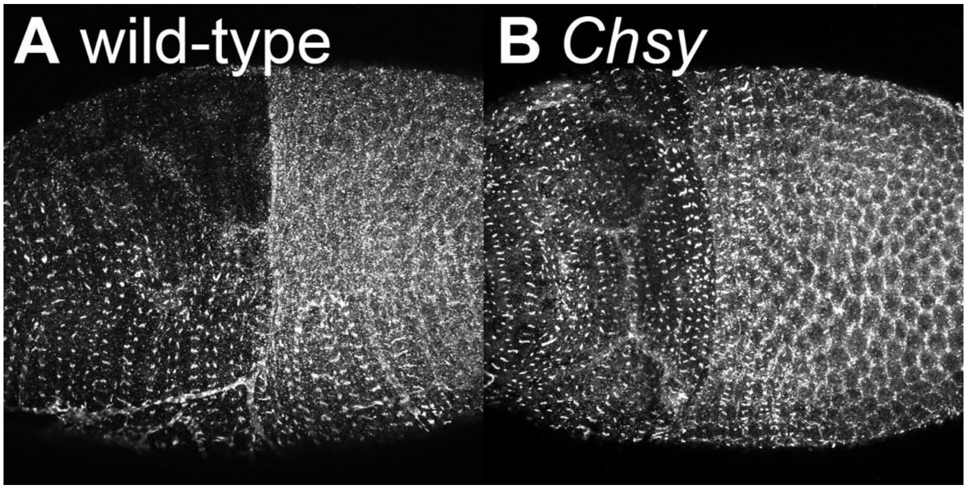

Fig. S3. Beta integrin staining of *Chsy* mutant egg chamber.
Stage 10 egg chambers of day-4 wild-type (A) and *Chsy* mutant (B) ovarioles were stained with anti-β integrin antibody. The muscle-muscle attachment sites can be visualized in the anterior (left) region.

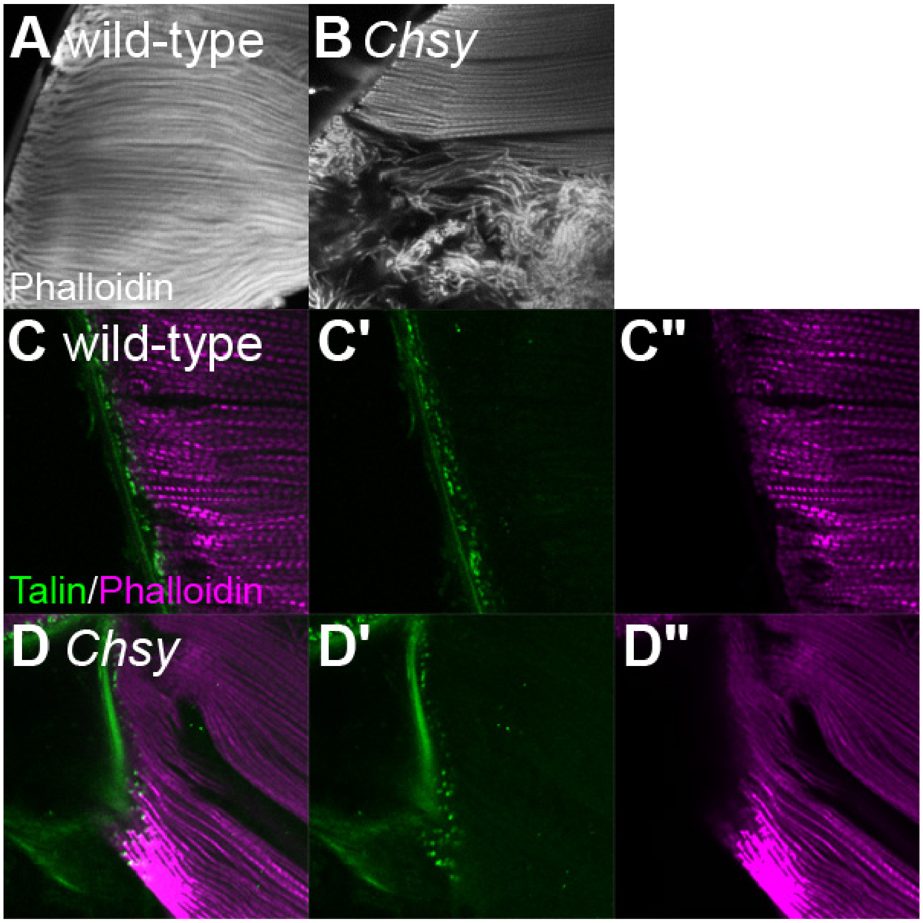

Fig. S4. Indirect flight muscle in *Chsy* mutant.

(A and B) Indirect flight muscles (IMFs) of day-3 wild-type (A) and *Chsy*^{2} mutant (B) adult females were stained with phalloidin. 67% of *Chsy*^{2} mutants show myofibril disarrangement in certain areas of the hemithorax (n=9). (C-D") IFM myotendinous junction was stained with anti-Talin (green) and phalloidin (magenta) in wild-type (C-C") and *Chsy*^{2} (D-D") adult females. No obvious defect in the myotendinous junction was observed in *Chsy* mutant.

Development • Supplementary information

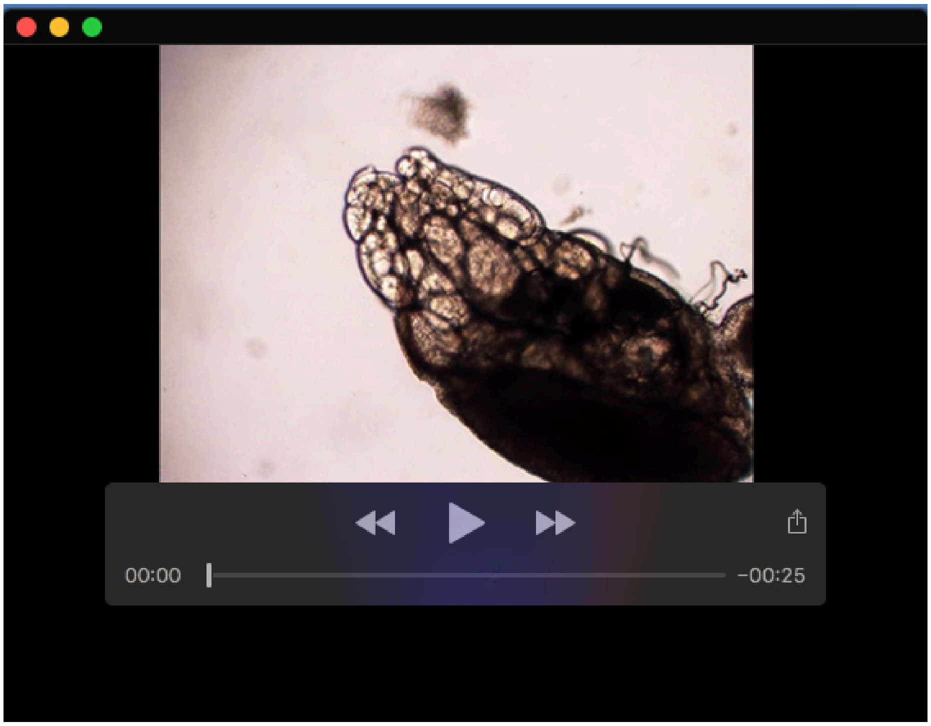

Movie 1. Live imaging of wild-type ovary showing normal rhythmic contraction.

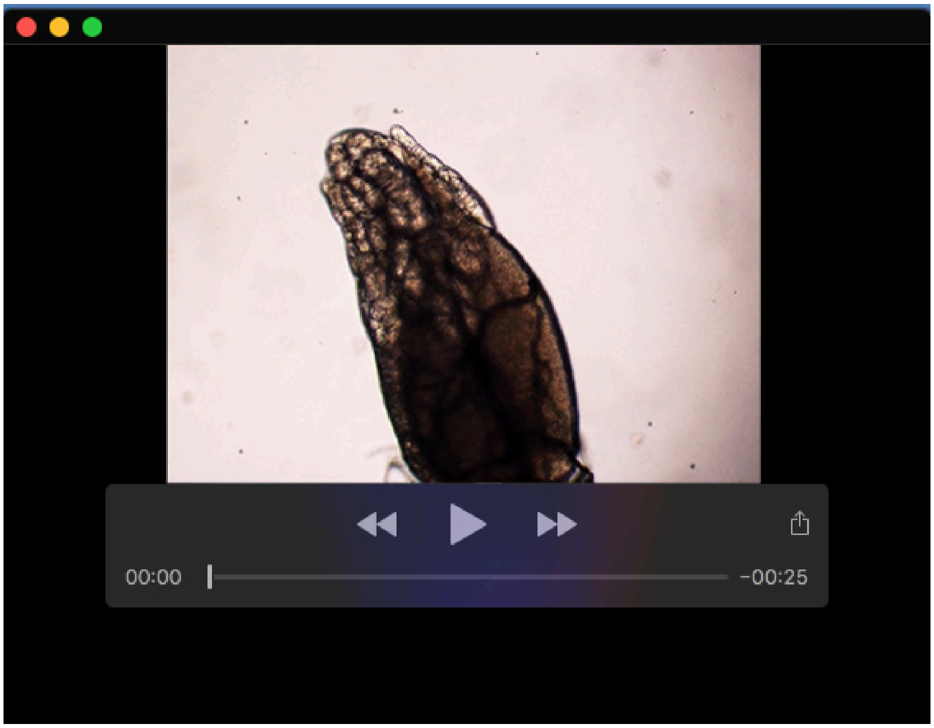

Movie 2. Live imaging of *Chsy2* mutant ovary showing disrupted contraction.
